# [^177^Lu]Lu-PSMA-617 Salivary Gland Uptake Characterized by Quantitative *In Vitro* Autoradiography

**DOI:** 10.3390/ph12010018

**Published:** 2019-01-24

**Authors:** Roswitha Tönnesmann, Philipp T. Meyer, Matthias Eder, Ann-Christin Baranski

**Affiliations:** 1 Department of Nuclear Medicine, University Medical Center Freiburg, Faculty of Medicine, University of Freiburg, 79106 Freiburg, Germany; roswitha.toennesmann@uniklinik-freiburg.de (R.T.); philipp.meyer@uniklinik-freiburg.de (P.T.M.); matthias.eder@uniklinik-freiburg.de (M.E.); 2 Division of Radiopharmaceutical Development, German Cancer Consortium (DKTK), partner site Freiburg, and German Cancer Research Center (DKFZ), 69120 Heidelberg, Germany

**Keywords:** PSMA-617, salivary gland uptake, prostate cancer, endoradiotherapy

## Abstract

Irradiation of salivary glands remains the main dose-limiting side effect of therapeutic PSMA-inhibitors, especially when using alpha emitters. Thus, further advances in radiopharmaceutical design and therapy strategies are needed to reduce salivary gland uptake, thereby allowing the administration of higher doses and potentially resulting in improved response rates and better tumor control. As the uptake mechanism remains unknown, this work investigates the salivary gland uptake of [^177^Lu]Lu-PSMA-617 by autoradiography studies on pig salivary gland tissue and on PSMA-overexpressing LNCaP cell membrane pellets. Displacement studies were performed with non-labeled PSMA-617 and 2-PMPA, respectively. The uptake of [^177^Lu]Lu-PSMA-617 in glandular areas was determined to be partly PSMA-specific, with a high non-specific uptake fraction. The study emphasizes that [^177^Lu]Lu-PSMA-617 accumulation in pig salivary glands can be attributed to a combination of both specific and non-specific uptake mechanisms. The observation is of high impact for future design of novel radiopharmaceuticals addressing the dose-limiting salivary gland irradiation of current alpha endoradiotherapy in prostate cancer.

## 1. Introduction

The therapy of metastatic, hormone-refractory prostate cancer poses a major clinical challenge. Treatment options are limited, rendering new developments and advances in therapy strategies of high clinical interest. The development of radioactive labeled prostate-specific membrane antigen (PSMA)-inhibitors suitable for endoradiotherapy has enabled a promising new form of therapy for metastatic, hormone-refractory prostate cancer. In particular, PSMA represents an attractive target structure and proved suitable for highly sensitive and specific nuclear medicine imaging and therapy as it is overexpressed in almost all prostate carcinomas with markedly increased levels in metastases [1,2,3,4,5]. Physiological expression has been shown to be significantly lower, and is limited to a few organs [1,6]. Clinical experience with PSMA-targeting positron emission tomography (PET), especially with [^68^Ga]Ga-PSMA-11 [7,8,9,10,11,12,13,14], in patients with recurrent prostate cancer show that lesions can be detected in almost all patients, in some cases with very low PSA levels. These findings often have a high impact on further therapeutic strategies [11,12].

When used therapeutically, radiation dose to organs with physiological PSMA expression might be dose-limiting and can thus minimize the therapeutic success of radiolabeled PSMA-inhibitors. In particular, renal and salivary gland uptake can be of concern, which, in the case of a therapeutic application, gives rise to relevant organ doses and possible side effects. With the development of PSMA-617 showing an improved and fast kidney excretion, a highly promising compound is already being clinically investigated for endoradiotherapy of prostate cancer with ^177^Lu or ^225^Ac [15,16,17]. In a first-in-man study with [^225^Ac]Ac-PSMA-617, two patients with advanced disease showed complete remission as confirmed by drop in PSA below the detection limit and a radiologic response in [^68^Ga]Ga-PSMA-11-PET. Alpha therapy did not result in hematologic toxicity or renal impairment. Nevertheless, strong accumulation of PSMA ligands in salivary glands was described in numerous papers, leading to considerable side effects. The salivary glands are significantly and partially irreversibly damaged, in particular during alpha therapy with ^225^Ac with a mean radiation dose of approximately 2.3 Sv/MBq compared to 0.7 Sv/MBq for kidneys and 0.05 Sv/MBq for bone marrow [18]. The resulting xerostomia leads to a significant impairment of the patients’ quality of life and thus represents a dose-limiting side effect for therapeutic use of radiolabeled small molecule PSMA-inhibitors. In contrast, PSMA-targeting antibodies such as J591 labeled with ^177^Lu show no significant uptake in salivary glands. Unfortunately, myelotoxicity appears caused by longer blood circulation of antibodies, making them unsuitable for PSMA-directed alpha therapy [19,20]. On the other hand, a reduction of the dose-limiting salivary gland uptake of small molecule PSMA-inhibitors has not yet been achieved by specific molecular design. Besides many rather non successful approaches towards reduction of salivary gland uptake such as local cooling or lemon juice [21,22], Baum et al. reported a 64% decrease in [^68^Ga]Ga-PSMA-11 uptake after multifocal, ultrasound-guided injections of botulinum toxin A in a parotid gland [23]. Although first approaches resulted in reduced salivary gland uptake of PSMA-targeting radioligands, the exact mechanisms of tracer accumulation, especially the ratio of specific to non-specific uptake in salivary glands, are not yet sufficiently understood. Salivary glands are known to physiologically express PSMA, which results in a PSMA-specific uptake of small molecule PSMA-inhibitors [24]. However, the detected strong salivary gland uptake of PSMA-inhibitors in clinical studies does not correlate with the rather low physiological PSMA-expression in that tissue. This is underlined by the fact that other physiologically PSMA-expressing organs like intestine, spleen and kidney show similar or even lower radiation doses after endoradiotherapy compared to salivary glands [18,25,26]. 

Therefore, the present study investigates the salivary gland uptake of [^177^Lu]Lu-PSMA-617 by autoradiography enabling visualization and quantification of tissue radioligand distribution. Therefore, specific and non-specific uptake of [^177^Lu]Lu-PSMA-617 were analyzed on pig salivary gland, as a close human homologue, in comparison to LNCaP membrane pellets expressing human PSMA as a positive control. This study gives a further insight into the salivary gland uptake of small molecule PSMA-inhibitors, which is crucial for the future development of novel PSMA-inhibitors with reduced salivary gland toxicity in endoradiotherapy of prostate cancer.

## 2. Results

### 2.1. Radiolabeling of PSMA-617

Radiolabeling of PSMA-617 with ^177^Lu resulted in high radiochemical yields > 95%. Thus, the output of labeling reactions was directly diluted with the appropriate buffer for further use in subsequent experiments (Radiochemical purity > 95%). The molar activity amounted to 48 GBq/µmol.

### 2.2. PSMA-Specific Binding and Saturation Analysis of [^177^Lu]Lu-PSMA-617 on Pig Salivary Gland Tissue and LNCaP Membrane Pellets

**Figure 1:**
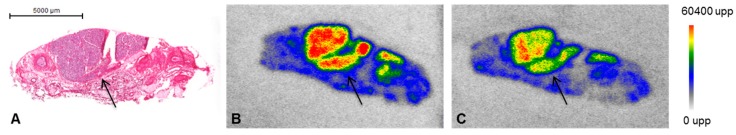
Salivary gland tissue cryosections (pig, 10 µm). Arrows indicate glandular areas. (**A**) H&E staining; autoradiography after incubation for 1.5 h at ambient temperature with 80 nM [^177^Lu]Lu-PSMA-617 showing total binding (**B**) and additional incubation with 80 µM 2-PMPA (highly potent PSMA-inhibitor) indicating non-specific binding (**C**).

The regional distribution of glandular areas could be visualized on 10 µm pig cryosections of salivary gland tissue after H&E staining (Figure 1A). In particular, glandular areas showed a high enrichment of [^177^Lu]Lu-PSMA-617 (Figure 1B). Additional blocking with 2-PMPA, a highly potent PSMA-inhibitor, resulted in a reduced uptake of radioligand in the glandular areas, whereby a relevant amount of non-specific bound [^177^Lu]Lu-PSMA-617 was still detectable with autoradiography (Figure 1C).

**Figure 2:**
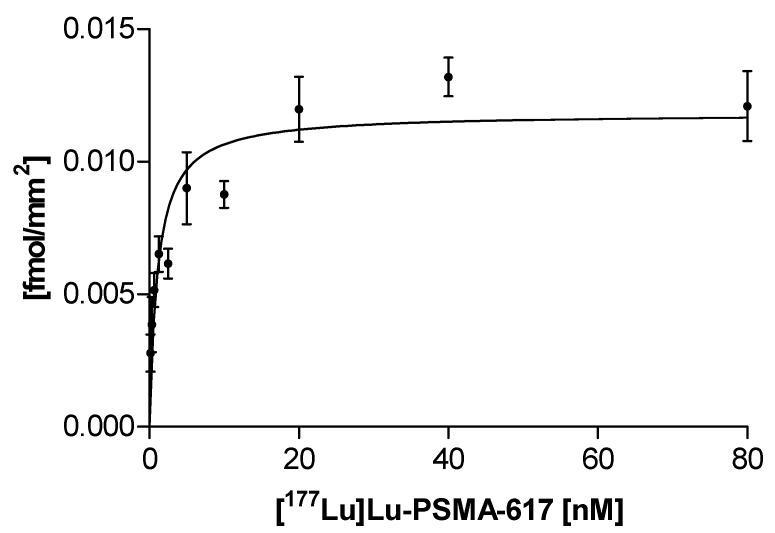
Saturation binding curve (specific binding) of [^177^Lu]Lu-PSMA-617 to pig salivary gland cryosections. Sections were incubated with 10 different concentrations of [^177^Lu]Lu-PSMA-617 (0.2–80 nM) for 1.5 h at ambient temperature. Autoradiography was performed with a Cyclone Plus Phosphorimager after an exposure time of 24 h.

For the saturation binding studies, LNCaP membrane pellets were used as a human PSMA expressing positive control. [^177^Lu]Lu-PSMA-617 revealed a *K*_d_ value in the low nanomolar range to both, pig salivary gland tissue (1.1 ± 0.2 nM) and LNCaP membrane pellets (2.0 ± 0.3 nM) (Figure 2, Table 1). 

Besides the specific binding of [^177^Lu]Lu-PSMA-617, non-specific uptake in salivary gland tissue accompanied by a reduced B_max_ value of 0.01 ± 0.0006 fmol/mm^2^ was notably high as compared to LNCaP membrane pellets (4.9 ± 0.5 fmol/mm^2^). Non-specific binding was determined at the presence of a 1000-fold excess of 2-PMPA, indicating a lower PSMA density and higher non-specific uptake in salivary gland tissue in contrast to PSMA overexpressing LNCaP membranes (Table 1). 

### 2.3. Competitive Binding Analysis of PSMA-617

**Figure 3:**
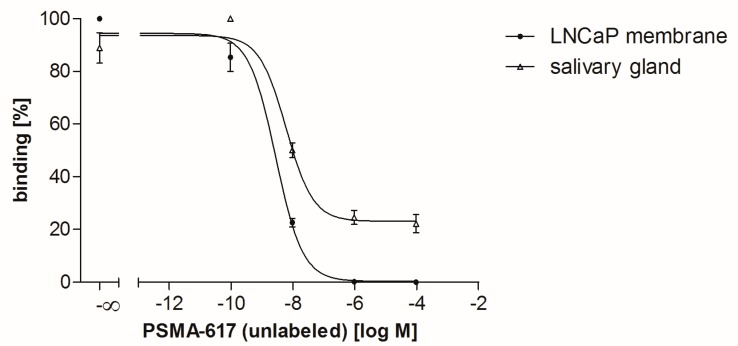
Competitive binding studies of PSMA-617 on LNCaP membrane pellets and pig salivary gland tissue. 6 nM radioligand ([^177^Lu]Lu-PSMA-617) were incubated with increasing concentrations (0.1 nM–100 µM) of PSMA-617 (unlabeled) as competitor for 1.5 h at ambient room temperature. Autoradiography was performed with a Cyclone Plus Phosphorimager after an exposure time of 48 h.

In competitive binding studies, PSMA-617 revealed high binding affinities in the low nanomolar range using the non-labeled precursor PSMA-617 as a competitor (Figure 3). The IC_50_ value on LNCaP membrane pellets was determined to be 2.7 ± 0.1 nM and on pig salivary gland tissue 6.0 ± 0.1 nM. Furthermore, a high non-specific uptake fraction in pig salivary gland tissue was detected, whereas LNCaP cell membrane pellets showed only negligible non-specific uptake (Figure 3).

## 3. Discussion

Endoradiotherapy of metastatic, hormone-refractory prostate cancer with small molecule PSMA-inhibitors represents a promising approach in the clinical challenging treatment regimen of those patients. In particular, endoradiotherapy with the small molecule PSMA-inhibitor PSMA-617 labeled with alpha emitters was reported to have the potential of complete remission in advanced stage prostate cancer patients [27]. Due to the high linear energy transfer of alpha emitters, critical doses are achieved in accumulating tissue. Besides a highly specific uptake in tumor tissue, rapid excretion of radiopharmaceuticals from non-target tissue is therefore crucial for a successful therapy and to minimize potential dose-limiting side effects. Kratochwil et al. reported the salivary gland toxicity to be the severe and the dose-limiting side effect during endoradiotherapy with ^225^Ac-labeled PSMA-617 strongly reducing the patient’s quality of life and limiting therapy success [18]. 

Thus, further advances in radiopharmaceutical design and therapy strategies are needed to reduce salivary gland uptake, thereby allowing the administration of higher doses potentially resulting in improved response rates and better tumor control. However, the molecular mechanism underlying the salivary gland accumulation of those radiopharmaceuticals still remains unknown. Several studies indicate that the uptake is potentially caused by a combination of non-specific and PSMA-specific uptake, whereby the exact ratio of specific to non-specific uptake is still undefined [23,28,29]. Therefore, the present study focused on the salivary gland uptake of PSMA-617 analyzed by autoradiography enabling an insight to binding characteristics. Pig salivary gland tissue was chosen as an easily accessible human homologue model representing the healthy organ. Glandular areas could be identified by H&E-staining allowing clear delimitation from surrounding tissue for precise quantification. As a PSMA-positive reference, cell membranes derived from LNCaP cells were analyzed, which are a well-established PSMA-expressing androgen-sensitive human prostate model. Saturation binding studies demonstrated that the uptake of [^177^Lu]Lu-PSMA-617 on pig salivary gland tissue is PSMA-specific, although a remarkably high proportion of non-specific uptake was noted. The 2-PMPA blocked uptake increased linear in dependence to the radioligand concentration on pig salivary gland tissue indicating a typical characteristic of non-specific binding. In contrast, LNCaP membrane pellets only showed a negligible non-specific bound fraction. Furthermore, the remarkably low B_max_ value detected in the saturation binding study indicates a low PSMA density on pig salivary gland tissue as compared to tumor cell membranes. This confirms that pig salivary gland tissue as a physiologically PSMA expressing tissue has a low PSMA density. As expected and in line with theoretical considerations, the binding affinity of PSMA-617 is not affected by the differences in PSMA densities. The *K_d_* values of [^177^Lu]Lu-PSMA-617 were observed to be in the low nanomolar range on both species, matching well with previously published binding affinities of PSMA-617 (K*_i_*: 2.34 ± 2.94 nM on LNCaP cells) [16]. The findings of the saturation binding study were confirmed with a competitive experimental design also revealing high binding affinities for PSMA-617 in the low nanomolar range on both tested species comparable to previously published binding data [16]. The respective competitive binding curves showed differences with regard to the bottom plateau representing non-specific binding. In contrast to LNCaP cell membrane pellets, a high non-specific bound fraction of PSMA-617 was observed again on salivary gland tissue samples. Both experimental set ups confirmed the previously published binding affinities of PSMA-617 on the tested species as well, however they were accompanied by a high non-specific bound fraction on pig salivary gland tissue.

The high amount of non-specific tracer uptake determined in this study might contribute to the phenomenon of strong salivary gland uptake of PSMA-inhibitors in clinical studies, which does not correlate with the rather low physiological PSMA-expression in that tissue [24]. In particular, non-specifically bound PSMA-617 was found to be located in glandular areas. Due to high blood supply, salivary glands are likely to enrich systemically applied pharmaceuticals. Ionic charges and molecular weight might also play an important role in non-specific radiotracer uptake. 

Further studies should focus on the rational design of chemically modified derivatives of clinically used PSMA-inhibitors in order to reduce the salivary gland uptake. In that context, concepts related to the non-specific uptake should be taken into account as well to improve the endoradiotherapy of prostate cancer. A more rapid elimination of the radiopharmaceutical from salivary gland tissue would avoid severe side effects and subsequently allow the administration of higher doses to better control the disease. 

## 4. Material and Methods

All commercially obtained chemicals were best grade and purchased from common suppliers. [^177^Lu]LuCl_3_ was purchased from ITG (Munich, Germany). PSMA-617 and 2-Phosphonomethyl pentanedioic acid (2-PMPA) were purchased from ABX (Radeberg, Germany). All reagents used in cell culture were purchased from Gibco©. 

### 4.1. Radiolabeling of PSMA-617 with ^177^Lu

The γ- and β-emitter [^177^Lu]LuCl_3_ (ITG, Munich), with a t_1/2_ of 6.7 days was used for the autoradiography studies. Radiolabeling was performed by adding 13 µL [^177^Lu]LuCl_3_ (~30–37 MBq) in 0.4 mM HCl, 7 µL of diluted peptide (0.1 mM solution of PSMA-617 in nanopure H_2_O) to 230 µL ammonium-acetate buffer (0.5 M, pH 5.4). The reaction mixture was incubated at 95°C for 30 min. After quality control by HPLC one equivalent of ^nat^Lu^3+^ (0.7 µL) was added and the mixture was incubated under the same reaction conditions. The radiochemical yield (RCY) was determined using high performance liquid chromatography (RP-HPLC; Chromolith RP-18e, 100 × 4.6 mm; Merck, Darmstadt, Germany). Analytical HPLC runs were performed using an Agilent 1200 series (Agilent Technologies, Santa Clara, CA, USA) equipped with a γ-detector. HPLC runs were performed using a linear gradient of A (0.1% trifluoroacetic acid (TFA) in water) to B (0.1% TFA in acetonitrile) (gradient: 5% B to 80% B in 15 min) at a flow rate of 2 mL/min. 

### 4.2. Quantitative In Vitro Autoradiography with [^177^Lu]Lu-PSMA-617

Pig salivary gland tissue was obtained from Prof. Dr. Mehrabi, University Hospital Heidelberg in cooperation with DKFZ, Heidelberg. Tissue was immediately frozen after surgical removal on dry-ice and stored at −80 °C in order to block further biological processes including protein degradation and tissue hardening.

The LNCaP membrane pellets were collected by washing LNCaP cells twice with ice-cold 0.05 M Tris-HCl (pH 7.4) in a first step. Afterwards the cells were scraped into ice-cold 0.05 M Tris-HCl (pH 7.4), collected by centrifugation, and homogenized using a Polytron PT1200E (Kinematica AG, Luzern, Switzerland) in the same buffer. After centrifugation at 120· *g* for 5 min at 4 °C, the supernatant was collected and centrifuged again at 48.000· *g* for 30 min at 4 °C. The resulting pellet was re-suspended in ice-cold Tris-HCl, transferred into a microfuge tube, and centrifuged at 20.000· *g* for 15 min at 4 °C. After withdrawal of the supernatant, the membrane pellet was stored at −80 °C. 

Tissue and membrane pellets were embedded in Tissue Tek (Tissue-Tek O.C.T., Sakura Finetek Europe B.V). Cryosections of 10 µm were prepared using a cryomicrotome (CM 1950, Leica Microsystems, Wetzlar, Germany) and mounted onto microscope slides (SuperFrost plus, Langenbrinck, Germany). Afterwards mounted sections were stored at least one day to improve adhesion of the tissue to the slide at −20 °C until quantitative *in vitro* autoradiography. 

Autoradiographic images were analyzed with a Cyclone Plus Phosphorimager (Perkin Elmer) and data analysis was performed with OptiQuant data processing software Version 5.0, Microsoft Excel and GraphPad Prism Version 5.01.

### 4.3. Saturation Binding Assay

For the determination of the dissociation constant (*K_d_*) and maximum binding capacity (B_max_) cryosections of salivary gland tissue (pig) and LNCaP membrane pellets were prepared for quantitative *in vitro* autoradiography as described above. 

Consecutive cryosections (20 per saturation binding assay) were incubated with 10 different concentrations of [^177^Lu]Lu-PSMA-617, one section to measure total binding and one section for non-specific binding, respectively. Samples were covered with 200 µL incubation solution containing increasing concentrations of [^177^Lu]Lu-PSMA-617 (0.2–80 nM) in 170 mM Tris-HCl buffer (pH 7.4) with 1% bovine serum albumin (BSA), bacitracin (40 µg/mL) and MgCl_2_ (5 mM) to inhibit endogenous proteases. Non-specific binding was determined at the presence of 2-PMPA at a final concentration of 80 µM. Sections were incubated for 1.5 h at ambient temperature. Thereafter sections were washed twice for 5 min in ice-cold 170 mM Tris-HCl buffer (pH 7.4) containing 0.25% BSA and once in ice-cold 170 mM Tris-HCl buffer (pH 7.4). Finally, sections were dipped in distilled water to remove buffer salts and dried rapidly under a stream of cool dry air. The sections were placed on a multisensitive storage phosphor screen for exposure in dedicated lead shielded cassettes. Exposure time for sufficient screen saturation was 10 min for LNCaP membrane pellets and 24 h for pig salivary gland tissue with the same experiment conditions. For data analysis the Phosphor Imager software (OptiQuant) expresses the radioactivity signal of the probes in digital light units per square millimeter (DLU/mm^2^). The intensity of the light from the retained energy is proportional to the amount of activity in the sample. As a standard, aliquots (2 µL) of the radioligand concentrations were spotted on ITLC paper (Polygram®SilG, Machery-Nagel, Düren, Germany) and co-exposed with the samples. From the known specific activity of the radioligand stock solution, the corresponding relative concentration (fmol/mm^2^) of the receptor was calculated. Regions of interests (ROIs) were drawn in the particular experiments to receive DLU/mm^2^ values. The dissociation constant (*K_d_*) and maximum binding capacity (B_max_) were analyzed and calculated by nonlinear regression using GraphPad Prism.

### 4.4. Competitive Binding Assay

In order to determine the potency (IC_50_) of PSMA-617 on salivary gland tissue (pig) and LNCaP membranes, a competitive binding assay was performed. Therefore, non-labeled compound PSMA-617 was tested with [^177^Lu]Lu-PSMA-617 as radioligand. For experiments, five adjacent cryosections were analyzed. Samples were covered with 200 µL incubation solution with increasing concentrations of the competitor ranging from 0.1 nM–1 µM in the presence of 6 nM radioligand. Sections were incubated for 1.5 h at ambient temperature, and were subsequently washed twice for 5 min in ice-cold 170 mM Tris-HCl buffer (pH 7.4) containing 0.25% BSA and once in ice-cold 170 mM Tris-HCl buffer (pH 7.4). Afterwards, sections were dipped in distilled water to remove buffer salts and dried rapidly under a stream of cool dry air. Autoradiography was performed as described in the section above. Exposure time for sufficient screen saturation was 48 h for all samples. Regions of interests (ROIs) were drawn using Phosphor Imager software (OptiQuant), which calculated the intensity units in each region as the fraction of activity in the region with the highest activity. IC_50_ values were analyzed by nonlinear regression using GraphPad Prism.

### 4.5. Statistical Aspects

All experiments were performed at least in triplicate. Quantitative data were expressed as mean ± SD. 

## Figures and Tables

**Table 1:** Binding affinity of [^177^Lu]Lu-PSMA-617 and receptor density of PSMA on LNCaP membrane pellets and pig salivary gland tissue.

Specimen	*K_d_* [nM]	Receptor Density (PSMA) B_max_ [fmol/mm^2^]
LNCaP membrane pellets (human, PSMA^+^)	2.0 ± 0.3	4.9 ± 0.5
Salivary gland tissue (pig)	1.1 ± 0.2	0.01 ± 0.0006
